# The effectiveness of e-learning in pediatric medical student education

**DOI:** 10.3402/meo.v21.29516

**Published:** 2016-02-10

**Authors:** Rima Khasawneh, Kari Simonsen, Jessica Snowden, Joy Higgins, Gary Beck

**Affiliations:** 1 Department of Pediatrics, University of Nebraska Medical Center, Omaha, NE, USA; 2 Children's Hospital, Omaha, NE, USA; 3 Office of Medical Education, University of Nebraska Medical Center, Omaha, NE, USA

**Keywords:** e-Learning, medical education, medical students, pediatrics

## Abstract

**Background:**

Electronic learning allows individualized education and may improve student performance. This study assessed the impact of e-modules about infection control and congenital infections on medical knowledge.

**Methods:**

A descriptive study was conducted involving third-year medical students on pediatric clerkship. e-Module content in three different formats was developed: a text monograph, a PowerPoint presentation, and a narrated PowerPoint lecture. Students’ use of the e-modules was tracked, as was participation in the infectious disease rotation and the order of pediatric rotation. Pre- and posttests specific to the e-module content and National Board of Medical Examiners (NBME) pediatric exam scores were recorded.

**Results:**

Among 67 participants, 63% of them visited at least one e-module. Neither accessing any e-modules, timing of pediatric clerkship, nor assignment to ID rotation resulted in improved posttest nor NBME scores. Seventy percent of students rated the e-modules as satisfactory and reported usage improved their confidence with the congenital infections topic.

**Discussion:**

e-Modules did not improve student performance on NBME or posttest; however, they were perceived as satisfactory and to have improved confidence among those who used them. This study underscores the importance of formally evaluating electronic and other innovative curricula when implemented within existing medical education frameworks.

Technology is increasingly being utilized in education to address the needs of learners. It has been demonstrated that e-learning promotes students’ individualized learning (1) and autonomy and reflective thinking (2) and allows self-pacing and flexibility (3). e-Learning has been shown to be at least as effective as live lecturing in many disciplines and across many levels of education, including undergraduate, graduate, and post-graduate education (1, 3–5). Learners’ satisfaction with e-learning varies, with reports of equal, increased, or decreased satisfaction compared with traditional lectures (6–10). We developed e-modules to teach principles of infection control and congenital infections to third-year medical students, as these topic areas were not emphasized in the general clerkship curriculum. We evaluated the impact of the e-modules on knowledge, satisfaction with the e-module curricula, and perceived confidence with the subject matter after e-module learning.

## Methods

A prospective, single-university study was conducted involving third-year medical students on pediatrics clerkship during the academic year 2013–2014. We designed e-modules on infection control and congenital infections, which were available as a detailed text monograph, PowerPoint slides with visual aids, and the same PowerPoint slides with a recorded narration. Modules were posted on Blackboard Learn^TM^, which tracked accession of the modules. Students who rotated on infectious diseases during their pediatric clerkship were recorded as they were more likely to be exposed to the e-module topics as part of their clinical experiences, and the pediatric clerkship end dates for students were tracked to determine if cumulative exposure to other medical rotations would impact knowledge. A 15-item, multiple choice pretest and self-assessment survey was provided. Students then could visit the e-modules. At the end of the clerkship, a different 15-item, multiple choice posttest with a satisfaction and confidence survey was administered. The overall National Board of Medical Examiners (NBME) scores were also collected. Pre/posttest data were analyzed using Wilcoxon rank sum test for dependent samples. Comparisons of pre- and post-surveys were calculated in aggregate as well as categorized by clerkship order and completion of the infectious diseases rotation. IBM SPSS version 17 software was used for analysis and considered a *p*-value<0.05 to be significant.

## Results

Of the total 126 eligible participants, 59 were excluded (incomplete data, *n*=49; incorrect test administered, *n*=10), leaving 67 participants for analysis. Of those, 67% accessed at least one of the e-modules. The rates of accession of different forms of each module (monograph, PowerPoint, and narrated PowerPoint) were 52, 57, and 63% for infection control and 60, 61, and 57% for congenital infection modules, respectively. The students’ performance on the posttest was significantly lower than the pretest for infection control (75% for pretest vs. 62% for posttest, *p<*0.005) but was unchanged for congenital infections e-module (58% pretest vs. 57% posttest, *p=*0.360). Accessing the e-modules did not have a significant impact on posttest scores (*U*=412.5, *p*=0.258) or on NBME scores (*U=*484, *p=*0.883). Neither rotation timing nor participation in the infectious disease rotation affected the posttest performance scores (chi-square=10.144, *p=*0.038; *U=*320, *p=*0.873). No significant correlation was found between NBME pediatric exam scores and pretest or posttest performance.

Students’ confidence with infection control was not improved after completing the modules (91% pretest vs. 76% posttest), while confidence was increased with congenital infections (37% pretest vs. 69% posttest). Seventy percent of students taking the infection control e-modules and 72% of those taking the congenital infection e-modules reported some or good overall satisfaction with the e-modules as a learning tool. Only one-third of the students found the traditional text-based monograph helpful for either topic.

## Discussion

Innovation in medical education is expanding, and as new electronic learning environments are introduced, evaluation and comparison with established curricula are growing imperatives. Although electronic learning has been shown to be at least as effective in knowledge acquisition (2), our study suggested there was no apparent preference for a particular e-module format based on accession rate. Accessing the modules did not have a significant impact on posttest performance nor on NBME scores, which indicates that modules did not improve medical knowledge.

In this study, neither the clerkship order nor participation in the infectious disease rotation affected the posttest performance scores or NBME performance. There was no significant correlation between NBME pediatric exam scores and pre- or posttest performance; thus, it is unlikely that students with a better preexisting fund of knowledge or test-taking skills would have confounded our evaluations. There was no improvement in students’ confidence regarding infection control following access of the e-modules. This could be due to better baseline knowledge of the topic, as these principles are part of medical practice in all other rotations. However, there was a substantial improvement in students’ confidence about approaching congenital infections after accession of the e-modules and fair overall satisfaction with the e-modules. The monograph was not perceived as helpful by the majority of participants, possibly due to time restrictions of medical students or the textbook-like format. Some of the limitations of our pilot study are the small sample size and the lack of direct comparison with traditional live lectures. Future directions include evaluation of modified e-module content, presentation format, and pre- and posttest tools to enhance student rotations and NBME performance through e-learning.

This study adds to the small but important body of literature demonstrating that e-modules are not a universally successful teaching tool and that their implementation in established curricula requires careful evaluation. Because of the unique design and confounders of each study based on the institution, e-modules deployed, assessments utilized, and target population of participants, it is difficult to compare our data directly with other studies, but it does illustrate the importance of evaluating the impact of e-modules in each setting to determine the usefulness of this approach.

## Conclusion

We conclude that e-modules for infection control and congenital infections were satisfactory to students and improved self-confidence in our sample of third-year medical students. However, there was no improvement in knowledge in either pre- and posttest scores or NBME exam results. As medical schools consider investing in e-learning initiatives, local examinations and NBME scores are metrics that can be utilized to inform curriculum committees about how to optimally integrate such modules into existing and future curricula. Further studies are needed for examination of e-modules, as their benefits may depend on the setting, subject matter, and delivery.

## Notes on Contributors

Rima Khasawneh, MBBS, is a fellow in Pediatric Infectious Diseases at the University of Nebraska Medical Center, Omaha, NE.

Kari A. Simonsen, MD, is an associate professor of Pediatric Infectious Diseases at the University of Nebraska Medical Center, Omaha, NE.

Jessica Snowden, MD, is an associate professor of Pediatric Infectious Diseases at the University of Nebraska Medical Center, Omaha, NE.

Joy Higgins, MS, is clerkship coordinator, Department of Pediatrics, University of Nebraska Medical Center, Omaha, NE.

Gary Beck, PhD, is director, Office of Medical Education, University of Nebraska Medical Center, Omaha, NE.

## Conflict of interest and funding

The authors have not received any funding or benefits from industry or elsewhere to conduct this study.
